# Creating concise and readable patient information sheets for interventional studies in Australia: are we there yet?

**DOI:** 10.1186/s13063-022-06712-z

**Published:** 2022-09-21

**Authors:** Tanya Symons, Joshua S. Davis

**Affiliations:** 1 Department of Medicine and Health, Northern Clinical School, The University of Sydney, Sydney, Australia; 2 Menzies School of Health Research, and Charles Darwin University, Casuarina, Northern Territory Australia; 3 Department of Infectious Diseases, John Hunter Hospital, Newcastle, Australia

**Keywords:** Informed consent, Interventional studies, Readability, Length, Comprehension, Risk perception, Low-risk trials

## Abstract

**Background:**

Participant information sheets and consent forms (PICFs) used in interventional studies are often criticised for being hard to read and understand. We assessed the readability and its correlates of a broad range of Australian PICFs.

**Methods:**

We analysed the participant information sheet portion of 248 PICFs. Readability scores were measured using three formulae: the Flesch Reading Ease, the Flesch-Kincaid Grade Level, and the Simple Measure of Gobbledygook (SMOG). We investigated how various features (including sponsor type and PICF type) correlated with PICF length and readability and examined compliance with other measures known to improve readability.

**Results:**

For a sample of 248 PICFs, the mean (standard deviation) Flesch Reading Ease score was 49.3 (5.7) and for the Flesch-Kincaid Grade Level 11.4 (1.1). The mean (SD) SMOG score was 13.2 (0.9). The median document length was 3848 words (8 pages). Commercial PICFs were more than twice as long as non-commercial, but statistically more readable (*p* = 0.03) when analysed using the SMOG formula. Subgroup analyses indicated that PICFs for self-consenters were statistically more readable than those for proxy consenters. The use of tables, but not the use of illustrations was associated with better readability scores.

**Conclusions:**

The PICFs in our sample are long and complex, and only 3 of the 248 achieved the recommended readability score of grade 8 or below. The broader use of best practice principles for writing health information for consumers and the development of more context-sensitive templates could improve their utility.

## Introduction

Informed consent is central to the ethical conduct of research, and participant information sheets and consent forms (PICFs) are a key component of the process. PICFs often contain complex scientific information. Well-written PICFs facilitate discussion, prompt questions, and support prospective participants’ understanding of a study’s nature and purpose, as well as its risks, benefits, and alternatives.

The National Health and Medical Research Council (NHMRC) in Australia has published the national templates for interventional studies and states that PICFs should be written in plain language (at school grade 8 equivalent or below) and should contain sufficient information for decision-making without being excessively long [1, 2]. Complex PICFs confuse rather than inform [3, 4], and, when long and legalistic, are less likely to be fully read [5–7]. Moreover, patients prefer shorter forms [8–11].

The latest quantification of literacy levels in Australia confirmed that 44% of Australians aged 15 to 74, rising to 65% for Australians aged 60 to 74, do not have literacy skills to meet the demands of daily life [12]. Literacy skills are measured in terms of reading levels, and ‘readability’ is how easy text is to read and understand [13]. Readability scores are one of a number of tools recommended to encourage the development of simpler, shorter, more appealing PICFs, which, combined, may improve a person’s understanding of the information presented [14–17].

Although several studies have examined the length and readability of PICFs, few are Australian-based, and those are small and limited to specific therapeutic areas [14, 18] or evaluated PICFs from a single source [3, 19]. Therefore, the authors conducted a national project to assess the length and readability of Australian PICFs. They also examined whether sponsor type and PICF type correlated with document length and readability scores and whether illustrations and tables improved these scores. Finally, as readability scores are only one indicator of how well a document reads, the authors examined the compliance with other best-practice measures to improve PICF readability.

## Methods

PICFs used between 2015 and 2020 for human interventional studies were obtained from a convenience sample of research organisations (32 were contacted with 21 providing PICFs) and from the Australian and New Zealand Clinical Trials Register. PICFs written for self-consenters or for proxy-consenters (parents/legal representatives) from all therapeutic areas and sponsor types were included. PICFs written for children or participants with learning disabilities, PICFs from non-interventional studies, and PICFs written in a language other than English were excluded. To maximise generalisability, PICFs were obtained from a convenience sample of organisations located in all Australian states and territories, including coordinating centres, industry sponsors, public and private hospitals, medical research institutes, trial networks, and research groups. To minimise selection bias, random samples of up to 25 PICFs were requested from research offices in large universities or teaching hospitals that typically host well over 25 interventional studies per year. These organisations were asked to select PICFs from their database using an online random number generator and a link to an online generator was provided. Organisations with fewer than 25 studies, typically trial networks, trial units, and individual research teams, were asked to provide all available PICFs.

A total of 289 were collected and coded by PICF type (self-consent versus proxy consent) and sponsor type (commercial versus non-commercial), study characteristics, illustrations, tables, and other elements related to document format, layout, and language use. Duplicates and ineligible PICFs were removed, leaving 278 PICFs. As we received a higher-than-anticipated response from non-commercial oncology networks/units, our sample of oncology PICFs substantially overrepresented oncology trial activity in Australia. Therefore, a random sample generator was used to select 30 non-commercial oncology PICFs for removal. A total of 248 PICFs were thus included in the analysis.

Consent forms were removed before the page, and word counts were recorded. Documents were then prepared for the calculation of readability scores. The online program ‘ReadablePro’ (formally ‘Readability-Score’) was used to calculate the readability scores [20]. PICFs were prepared in accordance with the program’s guidelines, including the removal of titles, headings, bulleted lists, tables, and any full stops embedded in the sentences.

Readability formulae are a widely accepted method for assessing the average comprehension of a text by an average reader [21]. For our analysis, we selected three well-established formulae. The primary outcome measure was the Flesch Reading Ease score [22], a continuous variable with potential scores of 0–100, where a higher score indicates easier readability. A score of between 70 and 80 is equivalent to a grade 8 reading level.

The secondary analysis was based on the Flesch-Kincaid Grade Level [23] and the Simple Measure of Gobbledygook (SMOG) [24] for the total sample and then for each group, with comparison. These measures estimate the years of education a person needs to understand a piece of writing. The Flesch formulae calculate the scores based on word and sentence length and are built into most word processing programs. The SMOG score is derived from the proportion of words with 3 or more syllables.

Regarding the rationale for using these formulae, the Flesch formulae are widely recommended in government health literacy and plain language guidance and built into word processing programs. The SMOG formula is also easily accessible and the most suitable for assessing health literature [25–27] unlike other formulae which test for 50–75% comprehension, SMOG tests for 100% comprehension. This is considered important for documents informing healthcare decisions, as these documents are not intended to be skim-read [27]. Consequently, SMOG tends to produce scores that are 1–2 grades higher than the Flesch formulae.

Both Flesch-Kincaid Grade Level and SMOG have a significant correlation with expert ratings of readability conducted by health literacy experts [28]. However, readability scores have their limitations as they do not measure factors such as cohesion between sentences, typography, and word choice [25, 28]. To extend our readability analysis, ten additional best practice measures for writing for consumers were selected from the NHMRC PICF guidance [1] and Australian Commission on Safety and Quality in Health Care (ACSQH) guidance [29, 30] and analysed each PICF for their presence. Although not research-specific, the ACSQH guidance documents were considered relevant as Australian hospital accreditation against ACSQH Standards now extends to its clinical trial activity. Three measures (words per sentence, sentences per paragraph, and the use of passive voice) were calculated by the ReadablePro program. To provide an objective measure for ‘word choice’, we selected seven complex words (listed in Table 3) where simpler alternatives are recommended in government guidance [31] and searched each PICF for inclusion of at least one of these words. These words were selected based on the likelihood that either they, or a simpler alternative, would be present in PICFs. For example, a PICF may state ‘*additional* blood’ will be taken, when ‘*extra* blood’ is the recommended alternative. Although the use of scientific terms or measurements is sometimes unavoidable, they should be explained. We searched PICFs for technical/medical terms or symbols used without a lay explanation that were likely to be unfamiliar to a lay audience (e.g. assay, subcutaneously, pharmacokinetics, peripheral vasodilatation, < 0.4/> 1.0 u/ml) or where simpler alternatives are recommended (e.g. biopsy, inflammation) [32].

Descriptive summary statistics (mean [SD] and median [IQR]) were used as appropriate.

Readability scores were near normally distributed, so Student’s *t*-test (unpaired) was used for comparison. Page and word count were non-normally distributed, so the Mann-Whitney *U* test was used for comparisons. All statistical analyses were performed using Stata version 15 (StataCorp, College Station, TX, USA), and *p* values < 0.05 were considered statistically significant.

## Results

Table 1 shows the study characteristics.

**Table 1:** Study characteristics

Parameter	Proportions: number (%)	
Type of trial	Randomised 214 (86%)	Non-randomised 34 (14%)
	Novel 129 (52%)	Established^a^ 119 (48%)
Intervention	Drug/device/biological 163 (66%)	Others 85 (34%)

^a^Includes studies involving repurposed therapies and diagnostics

**Fig. 1:**
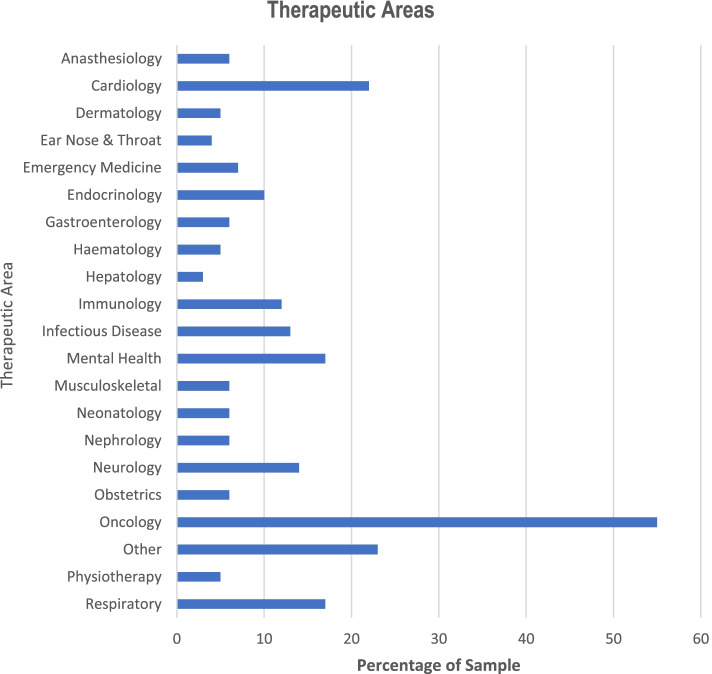
Therapeutic areas

Figure 1 illustrates the breakdown of studies by therapeutic area. Our sample reflects the national estimates of trial activity with oncology the therapeutic area with the greatest amount of trial activity. As expected, oncology trials dominate trial activity in Australia.

Table 2 shows the length and readability scores for the entire sample and by sponsor type.

**Table 2:** Length and readability of 248 PICFs according to sponsor type

	All (***n*** = 248)	Commercial (***n*** = 77)	Non-commercial (***n*** = 171)	***p*** (comm versus non-comm)
**Number of pages**	8 (6–15)	18 (14–22)	7 (5–9)	< 0.001
**Number of words**	3848 (2536–6874)	7826 (6720–9666)	3018 (1917–4258)	< 0.001
**Flesch-Kincaid Grade Level**	11.4 (1.1)	11.3 (0.9)	11.4 (1.1)	0.41
**Flesch Reading Ease**	49.3 (5.7)	50.1 (5.0)	49.1 (6.0)	0.15
**SMOG**	13.2 (0.9)	12.9 (0.7)	13.3 (1.0)	0.03

*PICF* Participant information sheet and consent forms, *SMOG* Simple Measure of Gobbledygook

*Length*: The included PICFs (after removal of the consent forms which were up to 5 pages long) ranged from 2 to 32 pages. More than 10% had 20 or more pages. The median length was 8 pages (3848 words). PICFs for commercial studies were more than twice as long (18 pages) as those for non-commercial studies (7 pages, *p* < 0.001).

*Readability scores*: The mean Flesch Reading Ease score was 49.3 which equates to text that is *difficult* to read. The mean Flesch-Kincaid Grade Level was 11.4, equating to a late secondary school reading level. The mean SMOG score was nearly two grades higher, at 13.2, equating to the expected reading level of a university student.

Commercial study PICFs had higher (better) readability scores than non-commercial PICFs, being non-significantly but numerically higher with the Flesch Reading Ease (50.1 vs 49.1) and a lower (better) Flesch-Kincaid formulae (11.3 vs 11.4), and significantly higher when using SMOG (12.9 vs 13.3, *p* = 0.03).

Table 3 describes the correlation of four key features of interest with readability measured using the Flesch Reading Ease formula (primary outcome). The inclusion of tables was significantly associated with improved readability, but illustrations were not. Readability scores for commercial and non-commercial PICFs did not differ significantly, but PICFs designed for self-consenters had significantly higher scores than those for proxy-consenters.

**Table 3:** Flesch Reading Ease scores for 248 PICFs according to key features of interest

Feature	Feature present (mean (SD))	Feature absent (mean (SD))	***p*** value
**Illustrations**	*N* = 38 50.0 (6.7)	*N* = 210 49.3 (5.5)	0.49
**Tables**	*N* = 99 50.6 (4.7)	*N* = 149 48.5 (6.2)	0.004
**Commercial PICFs**	*N* = 77 50.1 (5.0)	*N* = 171 49.1 (6.0)	0.22
**Self-consent**	*N* = 187 48.7 (5.7)	*N* = 61 51.6 (0.9)	< 0.001

*PICF* Participant information sheets and consent forms, *SD* standard deviation

Table 4 shows the proportion of PICFs that complied with best practice recommendations for document readability contained in NHMRC and ACSQH guidance. Overall, compliance was poor. Notably, only 1% of PICFs complied with the target reading grade level of ≤ 8.

**Table 4:** Compliance with best-practice recommendations for readability

Source of advice	Measure	Compliance (%)
NHMRC	Grade 8 readability level or below	1
	Use of tables	40
	Font size ≥ 11 points	94
ACSQH	Words/sentence < 20	45
	Sentences/paragraph < 3	93
	Limited passive voice use (< 10%)	100
	Word choice checked (simple words in place of complex ones)^a^	3
	Medical/scientific terms fully explained	21
	Left justified text	39
	Use of illustrations	15

*NHMRC* National Health and Medical Research Council, *ACSQH* Australian Commission on Safety and Quality in Healthcare

^a^Assessed for the presence of seven root words with simpler alternatives recommended in government plain language guidance—‘additional’, ‘approximate’, ‘concerning’, ‘reimburse’, ‘require’, ‘retain’, and ‘subsequently’

**Fig. 2:**
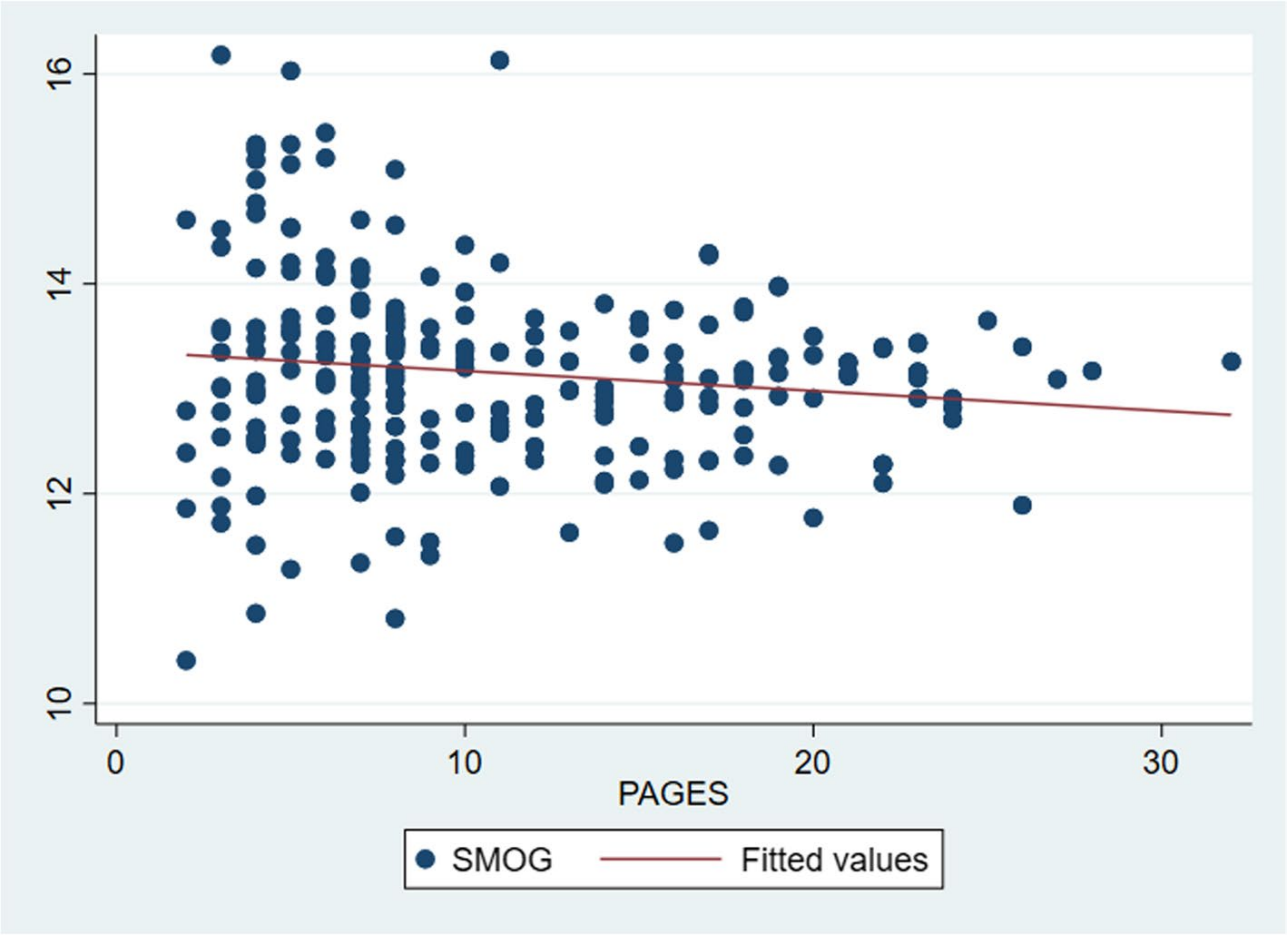
Scatterplot comparing the page count with the SMOG score in 248 participant information sheets (*r* = − 0.11, *p* = 0.08)

There was no correlation between the length of a document in pages and its readability (FRE; (Spearman’s rho = 0.05, *p* = 0.46) or the number of words in a document and the Flesch Reading Ease (Spearman’s rho = 0.03, *p* = 0.69). However, there was a non-significant negative correlation (*r* = − 0.11, *p* = 0.08) between the number of words in a document and its SMOG score (Fig. 2).

## Discussion

In this large convenience sample of Australian PICFs, very few PICFs met the reading level of grade 8 and the mean reading grade scores for grades 11 (Flesch Kincaid) and 13 (SMOG). Given that 44% of the adult population has a reading level below grade 11 [13], a large proportion of the population would have difficulty reading and understanding these PICFs.

There is evidence that in an educational context, people are unlikely to fully read documents that contain more than 1000 words [5]. The median length of our sample is over 3000 words, suggesting that many PICF may be too long to fully read. They also appear to be getting longer. Compared to the last Australian evaluation in 2014 [19], which analysed a similar proportion of commercial and non-commercial studies, the mean word count increased by 24%.

As commercial studies tend to involve novel interventions and/or unregistered drugs, it is not surprising their PICFs contain considerably more information. However, the finding that commercial PICFs are more readable was contrary to expectations. One possible explanation is that commercial sponsors may be more likely to use professional medical writers to develop their PICFs, while investigators tend to write these documents themselves.

The reason PICFs are easier to read when designed for self-consenters is unclear. Although this finding was in keeping with a prior analysis [33], our finding may simply be due to the higher proportion of non-commercial PICFs in the proxy-consenter cohort.

Our analysis confirms that compliance with best practice recommendations for plain language writing is patchy and appears to be dependent on whether the recommendation is reflected in the NHMRC PICF templates [1]. The use of tables was significantly associated with improved readability scores, and this design feature was effectively used in many PICFs, especially those with extensive safety information. Illustrations did not improve readability scores, perhaps because they were used to support text rather than replace it; however, their inclusion could improve the understanding in ways that cannot be detected by readability scores.

Despite their limitations, readability formulae are useful tools. Of the three formulae, SMOG has some key advantages. It is widely accessible and, as the only formula based on 100% expected comprehension, is most suitable for assessing documents in which patients must confirm that they have read and understood every word. Readability formulae, however, cannot assess the many other factors that contribute to a document’s utility, such as its design, layout and language choice, and other best-practice recommendation for writing in plain language should supplement their use.

To ensure a participant’s decision-making capacity is not overwhelmed by the sheer volume of information in a PICF, more imaginative ways are required to present this information. Countries with flexible consent policies have shorter PICFs. The UK RECOVERY trial, for example, a platform trial of therapeutics for COVID-19 (ClinicalTrials.gov: NCT04381936ISRCTN), has recruited more than 40,000 patients using a three-page information sheet that accurately reflects the risks of a study involving repurposed therapies. In the USA, the revised common rule requires researchers to consider what information a ‘reasonable person’ would want to have to decide whether to participate [34]. Conversely, Australian templates are criticised for their rigidity and focus on mitigating the risk of medico-legal exposure [35]. Some commentators suggest an excessive focus on risk can harm study participants through a phenomenon known as ‘the nocebo effect’ [36], when excessive risk information results in participants expecting side effects and thus experiencing side effects. Others suggest that legalistic PICFs can lead to the inappropriate rejection of studies due to an exaggerated perception of risk [37, 38]. Although one-size-fits-all templates can facilitate an ethical review, they can also inhibit the critical thinking needed to determine what content is most appropriate.

Another option would be to provide plain-language guidance on best practice principles for the development of PICFs, illustrated with examples of well-written PICFs or optional templates that consumers and researchers can use to co-design these critical documents.

Finally, the best way to confirm that a PICF is fit-for-purpose is to seek advice from the people it has been prepared for. In Australia, operational requirements and infrastructure are being implemented that encourage higher levels of consumer involvement in research which should enable greater levels of end-user involvement in the development of PICFs, advocated by commentators [10, 39].

The study’s strengths were the large sample size and the diversity of the PICFs sourced compared to previous studies. In addition, our assessment extended beyond readability scores to include several features recommended by governments and national health agencies to improve document performance.

Our study has several limitations. We provided organisations with written guidance on how to obtain a random sample of PICFs; we did not monitor this requirement. Our proportion of commercial PICFs (31%) was small compared with the national statistics for public health organisations (48% were commercial) [40]. This may have reduced the true estimate of PICF length in our combined analysis. Furthermore, although we confirmed that oncology dominates trial activity in Australia, we were unable to find precise estimates of activity, so even after reducing our sample, it may still be over representative. As oncology trial PICFs were significantly longer than other therapeutic areas, this may have led to an overestimation of PICF length. However, if this is the case, it would partially offset the underestimation of length due to the smaller than expected cohort of commercial PICFs. Another limitation is that the parameters assessed do not encompass the entire consent process. For example, a high-quality consent discussion is likely to contribute to participants’ understanding [41], and may well mitigate any inadequacy in the form itself. Finally, our analysis only evaluated written PICF documents without considering advances in electronic consent or the use of multimedia to improve the consent processes.

## Conclusion

Few Australian PICFs in our sample of interventional studies are written at a reading level the population can understand and most also contain considerably more information than a person is likely to fully read. Consequently, patients may miss an important detail, which diminishes the value of the PICF as an instrument to support informed decision-making.

The present study suggests there is a need for a more context-based approach to PICF development. Although ‘one-size-fits-all standard wording’ templates are comforting for both researchers and ethics committees, their rigid application can result in the description of risks being overinflated and patients inappropriately rejecting studies. Instead, PICF guidance could be revised to incorporate existing best practice principles for creating plain language health information, with templates or exemplary PICFs used to illustrate how context-sensitive documents could be written for various study types and risk levels. The knowledge that participants prefer simpler forms is surely reason enough to redouble efforts to improve the utility of PICFs.

## Data Availability

The dataset generated and analysed during the current study (the PDF reports of PICFs created by the ReadablePro program) are not publicly available as they contain proprietary information, and we do not possess the rights to share. However, a summary spreadsheet of all non-proprietary data is available from the corresponding author on request.
